# Transmembrane 4 L Six Family Member 1 Suppresses Hormone Receptor-–Positive, HER2-Negative Breast Cancer Cell Proliferation

**DOI:** 10.3389/fphar.2022.770993

**Published:** 2022-01-27

**Authors:** Jie Chen, Jin Zhu, Shuai-Jun Xu, Jun Zhou, Xiao-Fei Ding, Yong Liang, Guang Chen, Hong-Sheng Lu

**Affiliations:** ^1^ Department of Experimental and Clinical Medicine, Taizhou Central Hospital (Taizhou University Hospital), Taizhou University, Taizhou, China; ^2^ Department of Breast Surgical Oncology, Jiangxi Cancer Hospital, Nanchang, China,; ^3^ Graduate School of Medicine, Hebei North University, Zhangjiakou, China; ^4^ Department of Pathology, Taizhou Central Hospital (Taizhou University Hospital), Taizhou University, Taizhou, China

**Keywords:** transmembrane 4 L six family 1, breast cancer, hormone receptor–positive, HER2-negative, tumor suppressor

## Abstract

**Background:** The prognosis of breast cancer varies according to the molecular subtype. Transmembrane 4 L six family 1 (TM4SF1) exhibits different expression patterns among the molecular subtypes of breast cancer. However, the expression profile of TM4SF1 in hormone receptor HR^+^HER2^-^ breast cancer remains unclear.

**Methods:** TM4SF1 mRNA levels were examined in major subclasses of breast cancer by analyzing The Cancer Genome Atlas (TCGA) datasets. In addition, TM4SF1 protein and mRNA levels in HR^+^HER2^-^ breast cancer tissue samples were determined by immunohistochemistry and Western blot assay. The effect of TM4SF1 on cell proliferation was evaluated using MTT, colony formation, 3D organoid, and xenograft models, following the TM4SF1 overexpression or knockdown.

**Results:** TCGA database analysis demonstrated that TM4SF1 was downregulated in breast cancer compared with the healthy adjacent breast tissue. In addition, the expression of TM4SF1 in basal-like one and the mesenchymal TNBC tissue was higher than that of the healthy adjacent breast tissue. Other types, including the luminal androgen receptor–positive TNBC tissue, expressed lower levels of TM4SF1. Immunohistochemistry and real-time quantitative PCR assays demonstrated that the TM4SF1 protein and mRNA levels were downregulated in the HR^+^HER2^-^ breast cancer tissue compared with the healthy adjacent tissue. Moreover, the TM4SF1 overexpression reduced the viability of MCF-7 and ZR-75-1 breast cancer cells, whilst reducing the number of colonies and 3D-organoids formed by these cell lines. By contrast, TM4SF1 knockdown led to an increased MCF-7 cell proliferation. However, in the TNBC cell line, MDA-MB-231, TM4SF1 silencing reduced cell proliferation. *In vivo*, the TM4SF1 overexpression inhibited MCF-7 xenograft growth in a nude mouse model, which was associated with the downregulation of the Ki-67 expression, apoptosis induction, and inhibition of the mTOR pathway.

**Conclusion:** TM4SF1 is downregulated in HR + HER2-breast cancer, and the overexpression of TM4SF1 suppresses cell proliferation in this cancer subtype.

## Introduction

Breast cancer is the most common cancer among women worldwide, with variable prognosis depending on the molecular subtype. There are five main molecular subtypes of breast cancer that are based on the genes a cancer expresses: luminal A, luminal B, triple-negative/basal-like, HER2-enriched, and normal-like breast cancer (Harbeck and Gnant, 2017). Triple-negative breast cancer (TNBC) is hormone receptor (HR)–negative (estrogen receptor (ER)– and progesterone receptor (PR)–negative) and HER2-negative. This type of cancer is more common in women with BRCA1 gene mutations, grows and spreads faster, has limited treatment options, and is associated with poor prognosis (Nath et al., 2021). HER2-enriched breast cancer is HR-negative and HER2-positive. Anti-HER2 therapies are used to treat all stages of HER2-positive breast cancer, from the early stage to metastasis (Ocaña et al., 2020). Luminal B breast cancer is HR-positive and either HER2-positive or HER2-negative with high levels of Ki-67. This kind of cancer usually grows faster than luminal A breast cancers, which is HR-positive, HER2-negative, and has low levels of the protein Ki-67. Luminal A cancers are low-grade, tend to grow slowly, and have the best prognosis. Normal-like breast cancer is similar to luminal A disease. Endocrine therapy is usually indicated for ER^+^ breast cancer (Spring et al., 2016). However, resistance to this form of therapy is common and may lead to disease recurrence, metastasis, and eventually death (Xiao et al., 2018). Hormone receptor (HR)^+^HER2^-^ breast cancer remains the dominant contributor to annual breast cancer deaths worldwide (Cuyún Carter et al., 2021).

Transmembrane 4 L six family 1 (TM4SF1) is a 202-amino-acid protein of the TM4 superfamily, with four hydrophobic transmembrane domains (Fu et al., 2020). TM4SF1 mediates signal transduction events that play a role in the regulation of cell development, activation, growth, and motility. It is a cell surface antigen and is highly expressed in different carcinomas (Fu et al., 2020). TM4SF1 was initially observed to be upregulated in malignant melanoma in response to activated HERmrk kinase, a chimeric protein consisting of the extracellular part of EGFR (HER) and the cytoplasmic part of *Xiphophorus* melanoma receptor kinase (Teutschbein et al., 2010). TM4SF1 is also highly expressed in other cancer types, including prostate (Allioli et al., 2011), ovarian (Gao et al., 2019), glioma (Wang et al., 2015), colorectal (Park et al., 2017), liver (Zhu et al., 2021), thyroid (Lee et al., 2019), lung (Ma et al., 2018), pancreatic (Cao et al., 2016), and breast cancers (Tu et al., 2012; Xing et al., 2017; Fan et al., 2019). However, TM4SF1 also can be downregulated in gastric cancer (Peng et al., 2018) and mammary ductal carcinoma *in situ* (Abba et al., 2004).

The expression and function of TM4SF1 in breast cancer development are unclear and may depend on the molecular subtype. Indeed, it has been demonstrated that TM4SF1 is downregulated in mammary ductal carcinoma *in situ* (Abba et al., 2004). In addition, the overexpression of p23 in the MCF-7 breast cancer cell line results in increased invasion, which is associated with TM4SF1 downregulation (Simpson et al., 2010). These aforementioned studies indicate that TM4SF1 may act as a tumor suppressor in breast cancer. However, TM4SF1 is also positively correlated with cell migration, plays a major role in metastatic reactivation, and promotes relapse in TNBC (Gao et al., 2016).

Hormone receptor (HR)+HER2-breast cancer contributes to most breast cancer deaths (Spring et al., 2016). Clarifying the expression profiles of TM4SF1 in HR^+^HER2^-^ breast cancer and its role in this specific cancer progression is important for developing a potential therapeutic strategy for HR^+^HER2^-^ breast cancer. In the present study, The Cancer Genome Atlas (TCGA) database was used to characterize TM4SF1 expression profiles between different types of breast cancer. TM4SF1 expression profiles in HR^+^HER2^-^ breast cancer tissues were tested by immunohistochemistry and qRT-PCR assay; its roles in HR^+^HER2^-^ breast cancer development were investigated upon TM4SF1 overexpression or knockdown.

## Materials and Methods

### Cell Culture

MCF-7 was cultured in the MEM supplemented with 10% FBS and antibiotics. ZR-75-1 cells were cultured in the DMEM supplemented with 10% FBS and antibiotics. MDA-MB-231 cells were cultured in the RPMI-1640 medium supplemented with 10% FBS and antibiotics. 293T was cultured in the DMEM supplemented with 10% FBS and antibiotics. All cells were purchased from the Chinese Academy of Sciences Type Culture Collection. Cells were maintained at 37°C in a humidified atmosphere with 5% CO_2_.

### Immunohistochemistry

Human hormone receptor (HR)^+^HER2^-^ breast cancer tissue samples were obtained from Taizhou Central Hospital with the consent of all patients. The tissue samples were fixed in formalin and embedded in paraffin. Each slide containing both healthy adjacent breast tissue and tumor breast tissue samples was used for immunohistochemistry. The present study was approved by the Medical Ethics Committee of Taizhou Central Hospital (approval no. F-YXLL-004).

Tissue sections were rehydrated using xylene and graded concentrations of ethanol, incubated in sodium citrate (10 mM; pH 6.0) for 10 min, and then cooled down to room temperature. Endogenous peroxidase activity was then blocked with 3% hydrogen peroxide for 30 min at room temperature. The sections were permeabilized with 0.1% Triton and blocked in 10% goat serum for 30 min. The following primary antibodies were used: anti-TM4SF1 (1:400, Abcam), anti–Ki-67 (1:200, Cell Signaling Technology, Inc.), and anti-FLIP (1:400, Cell Signaling Technology, Inc.). All primary antibodies were diluted in 5% goat serum and added to the sections at 4°C overnight. The sections were then incubated with a biotinylated goat anti-rabbit antibody (1:200; BD Pharmingen; BD Biosciences). For detection, streptavidin–horseradish peroxidase and a DAB substrate kit (BD Pharmingen; BD Biosciences) were used. Counterstaining was carried out using hematoxylin.

### Lentiviral Plasmid Transduction

The pLenti-EF1a-EGFP-P2A-Puro-CMV-TM4SF13-Flag plasmid was designed and synthesized by OBiO Biotechnology Corp., Ltd. The GenBank accession number for the TM4SF1 gene is NM_014220.3. The pMDG2.G and psPAX2 plasmids for the lentivirus assembly were obtained from Addgene, Inc. The 293T cells stably expressing the TM4SF1 plasmid and a control vector were prepared by lentiviral transduction. Briefly, co-transfection was performed by combining the lentiviral plasmid (2.5 μg) with the packaging plasmids (0.75 μg pMD2. and 1.90 μg psPAX2). The 293T cells were then infected with viral supernatants containing 8 μg/ml polybrene (MedChemExpress). Transduced cells were selected using 1 μg/ml puromycin.

*Small interfering RNA (siRNA) transfection.* Synthetic siRNAs targeting TM4SF1 were purchased from Shanghai GenePharma Co., Ltd. The sequences were as follows: siRNA-1, 5′-GCA​CGA​TGC​ATC​GGA​CAT​TCT-3′; siRNA-2, 5′- GCT​ATG​GGA​AGT​GTG​CAC​GAT-3′; and control-siRNA, 5′-UUC​UCC​GAA​CGU​GUC​ACG​UTT-3′. siRNA was transfected using Lipofectamine®-RNAiMAX (Invitrogen; Thermo Fisher Scientific, Inc.).

### Reverse Transcription-Quantitative PCR

Total RNA was extracted using Trizol^®^ and then reverse-transcribed with the Prime Script™ RT reagent kit (Takara Biotechnology Co., Ltd.). The cDNA templates were amplified by qPCR using the PowerUp™ SYBR™ Green Master Mix (Thermo Fisher Scientific, Inc.). The primer sequences were as follows: TM4SF1 forward, 5′-TGC​AGG​ATC​TGG​CTA​CTG​TG-3′ and reverse, 5′-CAG​AAG​GTA​CTG​GCC​CTC​AG-3′; and GAPDH forward, 5′-GCA​CCG​TCA​AGG​CTG​AGA​AC-3′ and reverse, 5′-GCC​TTC​TCC​ATG​GTG​GTG​AA-3′. The thermocycling conditions were as follows: 1) 50°C for 120 s, 2) 95°C for 120 s, 3) 40 cycles of 95°C for 15 s and 60°C for 60 s, 4) 95°C for 15 s, 5) 60°C for 60 s, and 6) 95°C for 15 s. Gene expression levels were calculated using the ΔCq method. The mRNA levels of TM4SF1 were normalized to those of GAPDH mRNA.

### Immunoblotting

Immunoblotting was conducted using standard procedures. Briefly, the cells were resuspended in the RIPA lysis buffer (Beyotime Institute of Biotechnology). The sample was transferred to a 1.5-ml centrifuge tube and then centrifuged at 10,000 x g for 5 min at 4°C to pellet the cell and tissue debris and collect the protein lysates. Equal masses of protein (50 μg) were analyzed by SDS-PAGE. Antibodies against TM4SF1 (#ab113504, Polyclonal, Abcam, Cambridge, United States), AKT (#9272, Polyclonal), phosphorylated AKT (#9275, Polyclonal), and *β*-actin (#5125s, Polyclonal) (Cell Signaling Technology, Boston, United States) were obtained. Antibodies against Bax (BA0315), Bcl-2 (BA0412), caspase 3 (BM4340), caspase 9 (BM4619), and FLIP (M01295, Monoclonal, 7F10) were obtained from Boster Biological Technology (Wuhan, China). Antibodies against GAPDH (0411, Polyclonal), LC3 (G-4, sc-398822), and Beclin-1 (E-8, sc-48341) from Santa Cruz Biotechnology (Santa Cruz, CA) were used. All primary antibodies were polyclonal. Proteins were visualized using a peroxidase-conjugated secondary antibody (Sigma-Aldrich; Merck KGaA) using ECL solution (Thermo Fisher Scientific, Inc.) for detection.

### MTT Assay

Cell proliferation was evaluated using MTT assays. Briefly, 1 × 10^3^ cells were plated in a 96-well plate using a complete culture medium. After 4, 24, 48, or 72 h, MTT was added to each well at a final concentration of 0.5 mg/ml. The reaction was allowed to proceed for 4 h at 37°C. The formazan crystals were then dissolved in 100 μL DMSO. The plates were incubated in an orbital shaker at room temperature for 15 min, and the absorbance was then read at 490 nm. All assays were performed in triplicates.

### 3D Organoid Formation

Briefly (Chen et al., 2020), a total of 1 × 10^3^ cells were gently mixed with 30 μL Matrigel and plated as a droplet containing the single cells onto a prewarmed (in a 37°C incubator) 24-well ultra-low attachment plate. The droplets were allowed to solidify at 37°C for 15 min, then covered with 500 μL complete cell culture medium, and incubated at 37°C for 15 days 3D organoid images were examined under a microscope.

### Colony Formation Assay

For colony formation assays, 1 × 10^3^ cells were seeded into 6-well plates and incubated for 10 days at 37°C with 5% CO_2_. The colonies were fixed in 100% methanol, then stained for 30 min in crystal violet, and washed in ddH_2_O. The clones were counted under a microscope.

### Annexin V/Propidium Iodide Staining

Cell apoptosis was analyzed by flow cytometry. Following different treatments, cells were trypsinized and washed with PBS twice. The cells were then stained with Annexin V-FITC/propidium iodide (Beyotime Institute of Biotechnology) according to the manufacturer’s instructions. Apoptosis was analyzed by MultiCycle software.

### *In Vivo* Xenograft Model and Tumorigenicity

All animal experiments were approved by the Medical Ethics Committee of the Taizhou University College of Medicine (approval no. TZYXY 2020). A total of 24 male BALB/C-nu/nu mice (age, 6-8 weeks; average weight, ∼25 g) were obtained from Changzhou Cavens Animal Co., Ltd. The mice were housed in sterile cages under laminar airflow hoods at 20°C in a specific pathogen-free environment with a 12-h light/dark cycle and provided with autoclaved chow and water *ad libitum*. A total of 10^7^ parallel-controlled or TM4SF1-overexpressing MCF-7 cells were injected subcutaneously into the flank of the mice, respectively. Tumor dimensions were measured with calipers twice a week, and the volumes were calculated as (width)^2^ x length/2. After 12 weeks, the animals were sacrificed by cervical dissociation, and the tumors were removed and weighed. The largest tumor diameter was 1.434 cm, and none of the animals developed multiple tumors.

### Statistical Analysis

The data are presented as the mean ± standard deviation and were analyzed using Student’s unpaired *t*-test or one way ANOVA by GraphPad Prism software (GraphPad Software). A *p*-value < 0.05 was considered significant.

## Results

### TM4SF1 Expression Profiles in Breast Cancer by TCGA Database Analysis

**FIGURE 1:**
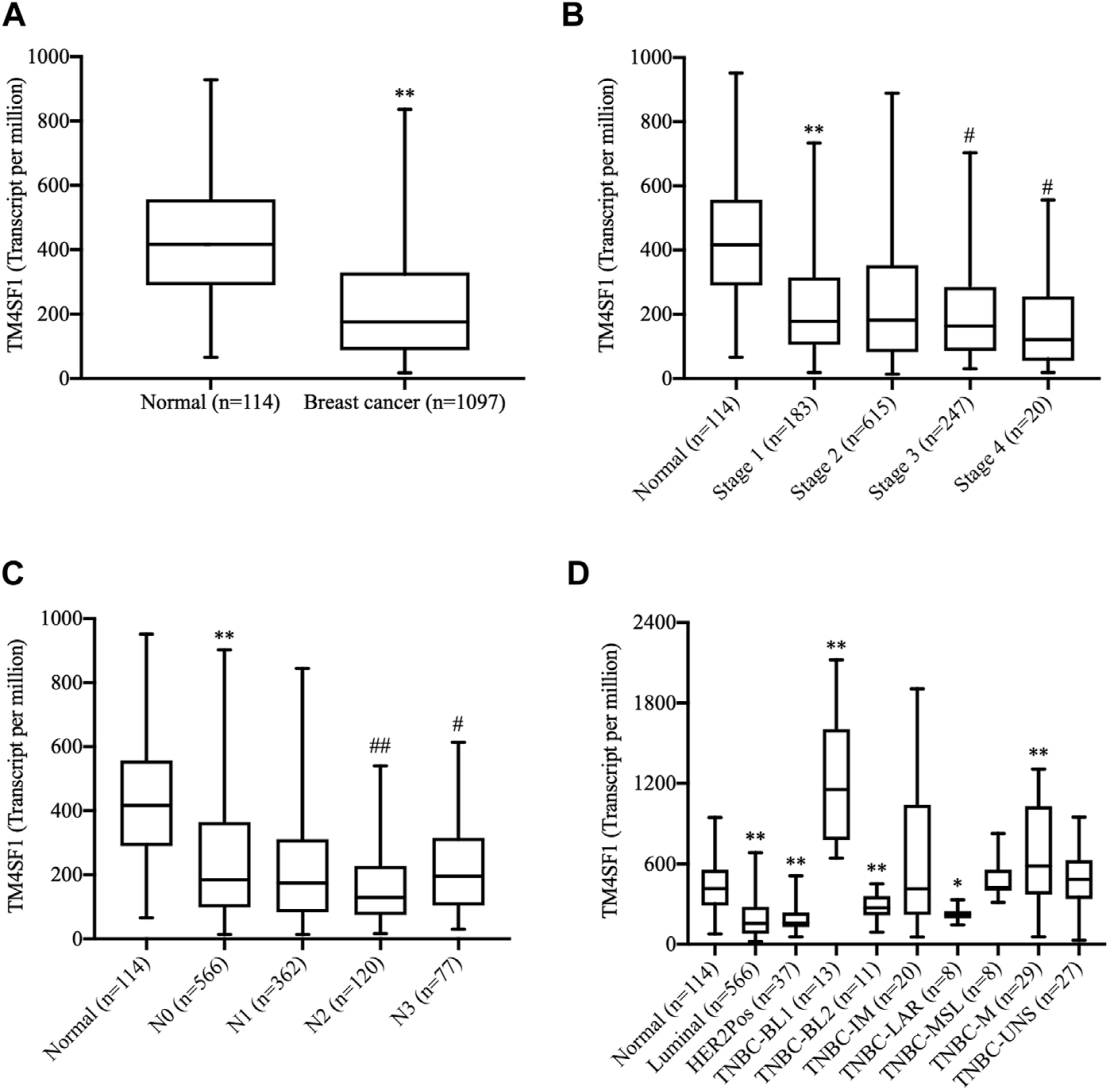
Analysis of the TM4SF1 expression in breast cancer using data from The Cancer Genome Atlas. **(A)** Unpaired analysis showed a significant decrease of TM4SF1 expression levels in the breast cancer tissue compared with the normal breast tissue. ^**^ *p* < 0.01 vs. the normal tissue. **(B)** Expression of TM4SF1 in BRCA based on individual cancer stages. ^**^ *p* < 0.01 *vs*. the normal tissue. ^##^ *p* < 0.01, ^#^ *p* < 0.05 *vs*. Stage 1. **(C)** Expression of TM4SF1 in BRCA based on the nodal metastasis status, ^**^ *p* < 0.01 *vs*. Normal, ^##^ *p* < 0.01, ^#^ *p* < 0.05 *vs*. N0. N0: No regional lymph node metastasis, N1: Metastases in 1–3 axillary lymph nodes, N2: Metastases in 4–9 axillary lymph nodes, N3: Metastases in 10 or more axillary lymph nodes. **(D)** Paired analysis revealed that TM4SF1 expression profiles in various molecular subtypes of breast cancer. ^*^ *p* < 0.05, ^**^ *p* < 0.01 *vs*. the normal tissue (ANOVA). TM4SF1, transmembrane 4 L six family 1; TNBC, triple-negative breast cancer; BL1, basal-like 1; BL2, basal-like 2; IM, immunomodulatory; M, mesenchymal; MSL, mesenchymal stem-like; LAR, luminal androgen receptor; UNS, unspecified.

The expression of TM4SF1 was first examined in breast cancer (*n* = 1,097) and normal breast (*n* = 114) TCGA data. TM4SF1 mRNA was expressed at lower levels in breast cancer than in normal tissue samples (Figure 1A). Moreover, TM4SF1 exhibited a lower expression along with the tumor stage (Figure 1B) and lower in lymph node–positive tissue specimens than that in lymph node–negative tissue specimens (Figure 1C). In addition, the expression levels of TM4SF1 were also analyzed in TNBC subclasses. Basal-like one and mesenchymal TNBC tissue expressed higher levels of TM4SF than the normal breast tissue. However, other types of breast cancer, including luminal androgen receptor–positive TNBC, expressed lower levels of TM4SF (Figure 1D). These findings indicated that the expression profiles of TM4SF1 in breast cancer varied with breast cancer subtypes.

### Downregulation of TM4SF1 in HR^+^HER2^-^ Breast Cancer

**FIGURE 2:**
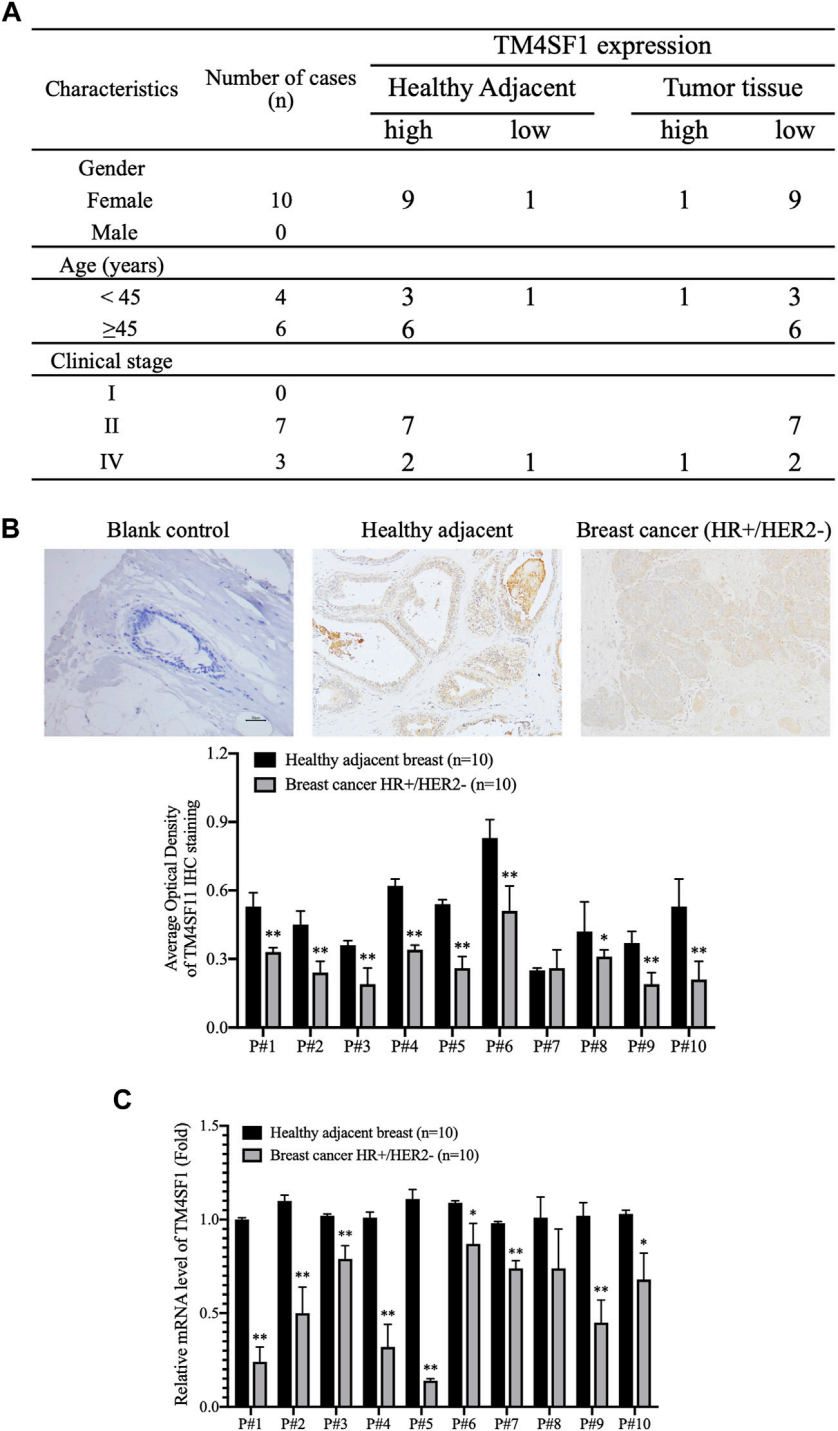
TM4SF1 expression profile in hormone receptor–positive, HER2-negative invasive breast cancer. **(A)** Clinicopathological characteristics of patients with breast cancer. *n* = 10. **(B)** Representative immunohistochemical staining images of TM4SF11 in breast cancer and healthy adjacent tissue sample (upper panel). Semi-quantification of TM4SF1 staining using the average optical density measured by ImageJ d 1.47 software (lower panel). mean ± SD, *n* = 10, ^**^ *p* < 0.01 compared with the healthy adjacent breast tissue by *t*-test analysis. **(C)** Data are presented as a fold change relative to the TM4SF1 mRNA levels in the healthy adjacent breast tissue (mean ± SD, *n* = 10). ^**^ *p* < 0.01 compared with the healthy adjacent breast tissue by *t*-test analysis. TM4SF1, transmembrane 4 L six family 1.

The expression of TM4SF1 was subsequently examined in HR^+^HER2^-^ patients with breast cancer by immunohistochemistry (*n* = 7; Figure 2A). TM4SF1 staining was semi-quantified by measuring the average optical density by ImageJ software. The results demonstrated that TM4SF1 protein levels were downregulated in the HR^+^HER2^-^ breast cancer tissue compared with the healthy adjacent breast tissue (Figure 2B) Moreover, total RNA was extracted from those above 7 samples, and the mRNA levels of TM4SF1 were determined using RT-qPCR. The TM4SF1 mRNA expression level was downregulated in the HR^+^HER2^-^ breast cancer tissue compared with that of the healthy adjacent breast tissue (Figure 2C).

### TM4SF1 Overexpression Inhibits MCF-7 and ZR-75-1 Cell Viability and Proliferation

**FIGURE 3:**
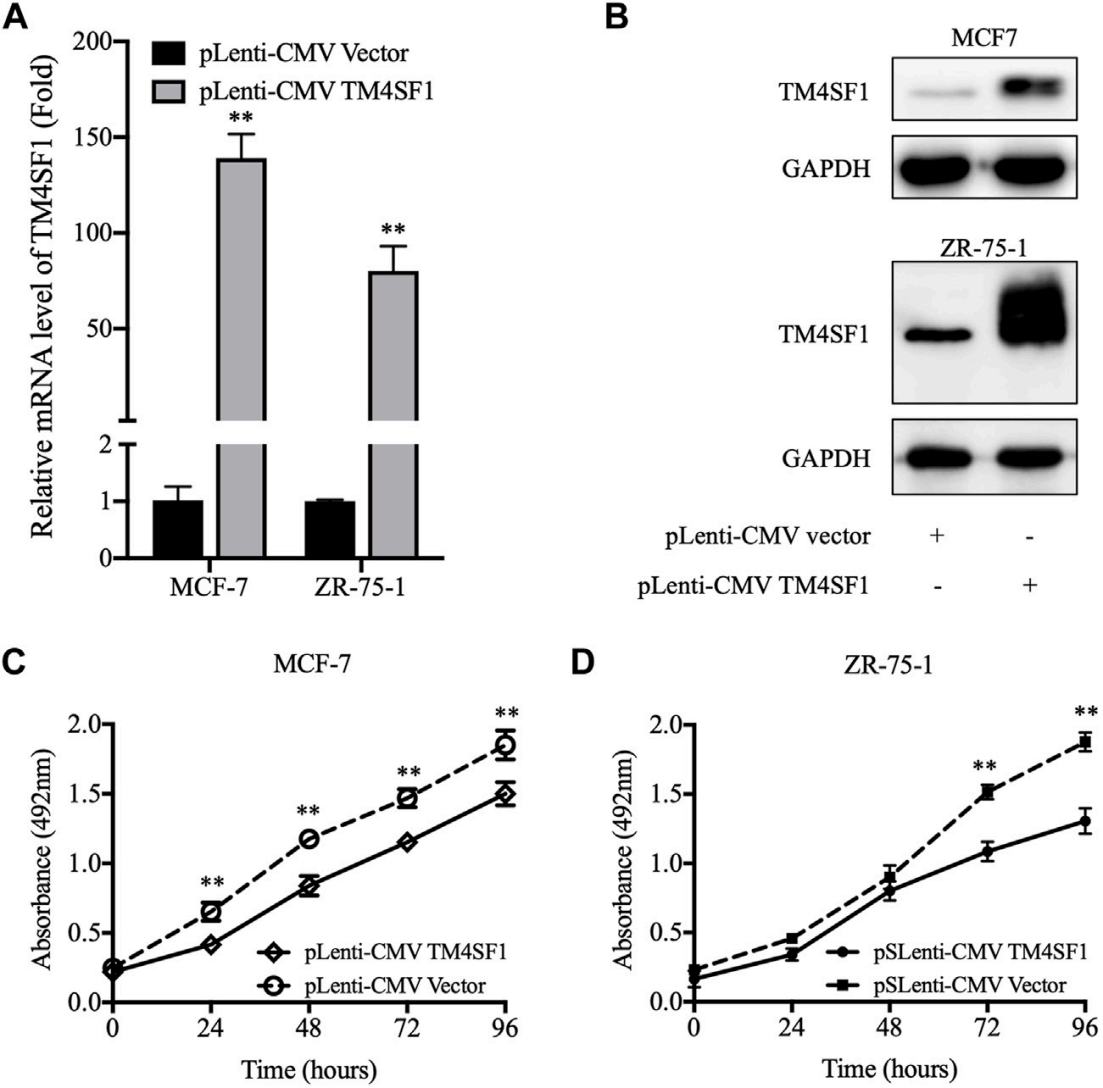
pLenti-CMV TM4SF1 transfection-mediated upregulation of TM4SF1 reduces cell viability. **(A)** TM4SF1 overexpression efficiency was evaluated using real-time quantitative PCR (normalized to GAPDH). Data are presented as fold changes relative to the TM4SF1 levels in control cells (mean ± SD, *n* = 3). ^**^ *p* < 0.01 compared with the pLenti-vector transfected group by *t*-test analysis. **(B)** TM4SF1 protein levels were significantly upregulated following with the TM4SF1 plasmid. GAPDH was used a loading control. **(C, D)** MTT assays were performed to detect the MCF-7 and ZR-75-1 cell viability (mean ± SD), ^*^ *p* < 0.05, ^**^ *p* < 0.01 compared with the pLenti-vector transfected group by *t*-test analysis. Data shown are representative of at least two independent experiments. TM4SF1, transmembrane 4 L six family 1.

The next experiments were designed to examine whether TM4SF1 acted as a tumor suppressor in HR^+^HER2^-^ breast cancer. TM4SF1-overexpressing cells were generated using the pLenti-CMV TM4SF1 lentiviral transduction system in the HR^+^HER2^-^ MCF-7 and ZR-75-1 breast cancer cell lines. As shown in Figures 3A,B, TM4SF1 mRNA and protein levels were markedly increased in MCF-7 and ZR-75-1 cells transfected with pLenti-CMV TM4SF1. Moreover, TM4SF1-overexpressing cells displayed reduced viability in an MTT assay compared with pLenti-CMV vector transfected cells (Figures 3C,D).

**FIGURE 4:**
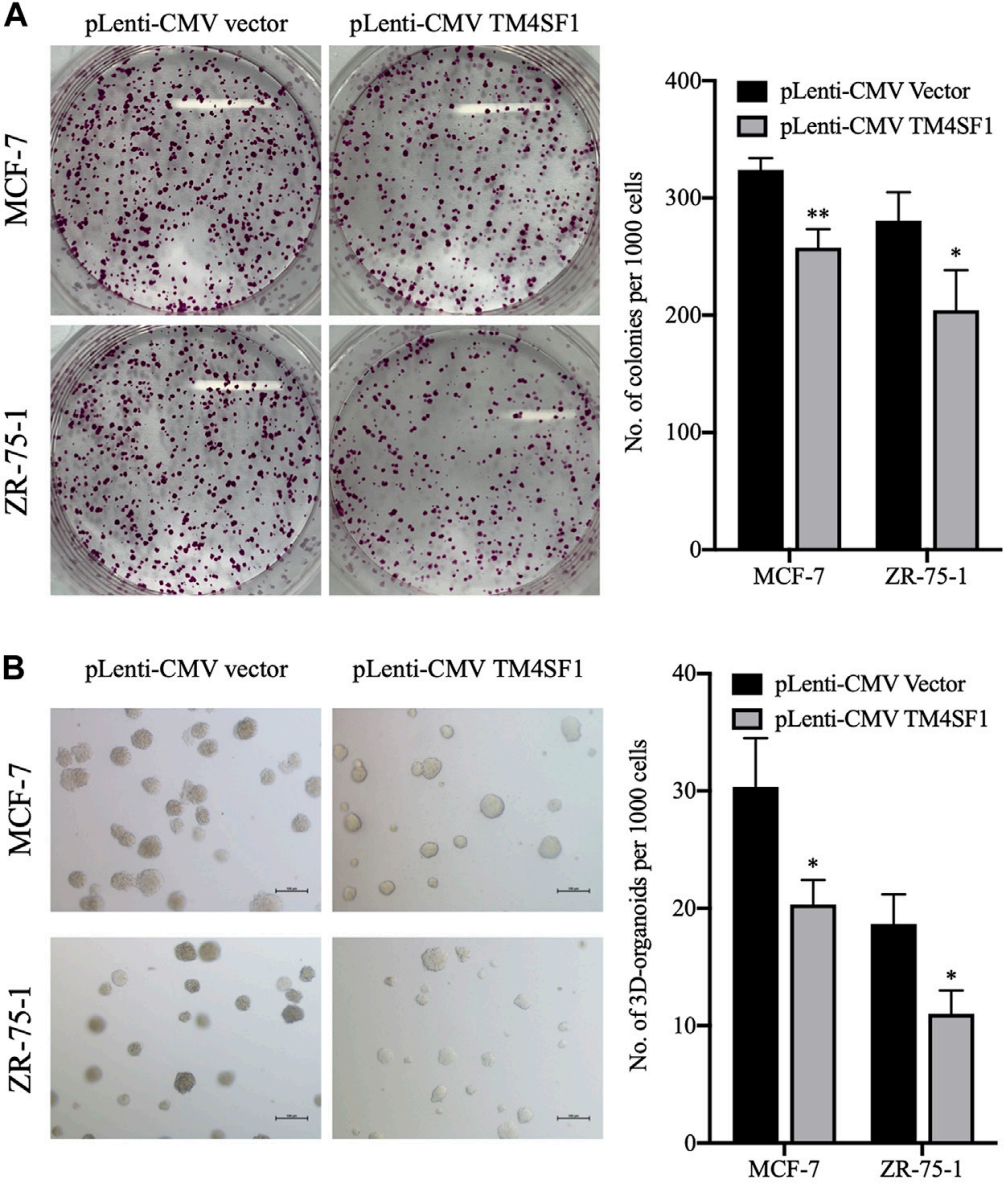
TM4SF1 overexpression inhibits colony and 3D spheroid formation in MCF-7 and ZR-75-1 cells. **(A)** Representative images of the colonies at least three independent experiments (left). Quantification data counting numbers (right; mean ± SD). ^*^ *p* < 0.05, ^**^ *p* < 0.01 compared with the pLenti-vector transfected group by *t*-test analysis. **(B)** Representative images of invasive extensions from the 3D spheroids from least three independent experiments. Objective, ×20. Quantification data counting numbers (right; mean ± SD). ^*^ *p* < 0.05 compared with the pLenti-vector transfected group by *t*-test analysis.

As the repopulation of residual tumor cells can lead to cancer recurrence, which depends on the ability of cells to divide (Matsuda et al., 2021), the effect of TM4SF1 on clonogenicity was evaluated using a colony formation assay. As shown in Figure 4A, the TM4SF1 overexpression significantly decreased the plating efficiency (colonies number from 1000 cells) after 10 days of incubation compared with the pLenti-CMV vector group (32 *vs*. 26% in MCF-7 cells; 27.5 *vs.* 19.5% in ZR-75-1 cells).

3D organoid models allow the understanding of complex biology in cancer development, whereas 2D models have not proven as successful. When performing 3D cell culture experiments, the cell environment can be manipulated to mimic that of a cell *in vivo* and provide more accurate data about cell-to-cell interactions, tumor characteristics, drug discovery, metabolic profiling, stem cell research, and other types of diseases (Jensen and Teng, 2020). Thus, the effect of TM4SF1 on breast cancer cell 3D organoid formation was then evaluated. MCF-7 and ZR-75-1 cells transfected with the pLenti-CMV vector could form ∼30 and ∼18 3D organoid structures per 1,000 cells, respectively. By contrast, TM4SF1-overexpressing MCF-7 and ZR-75-1 cells formed only ∼20 and 10 3D organoids per 1,000 cells, respectively (Figure 4B).

### TM4SF1 Knockdown Promoted MCF-7 Cell Proliferation *In Vitro*

**FIGURE 5:**
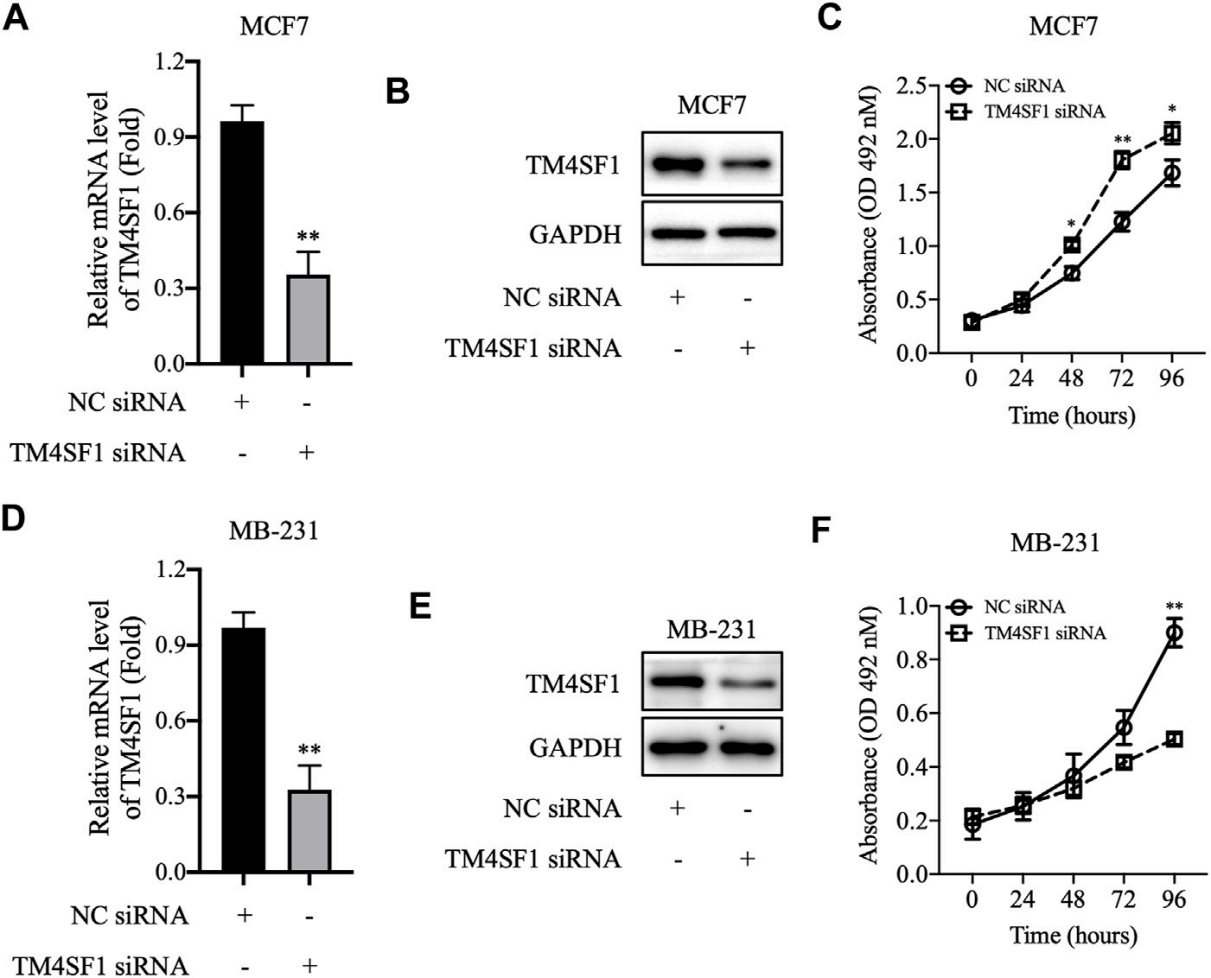
TM4SF1 knockdown in breast cancer cells. **(A–C)** TM4SF1 knockdown efficiency was evaluated using quantitative PCR (normalized to GAPDH). Data are presented as fold changes relative to the TM4SF1 levels in control cells, mean ± SD. ^**^ *p* < 0.01 compared with the negative control siRNA-transfected group by *t*-test analysis. **(B–E)** TM4SF1 protein levels were significantly downregulated following transfection with TM4SF1 siRNA. GAPDH was used as a loading control. **(D–F)** Effect of TM4SF1 siRNA transfection on cell proliferation, mean ± SD, ^*^ *p* < 0.05, ^**^ *p* < 0.01 compared with the negative control siRNA-transfected group by *t*-test analysis. Data shown are representative of at least two independent experiments. TM4SF1, transmembrane 4 L six family one; siRNA, small interfering RNA.

We further downregulated the TM4SF1 expression level by siRNA transfection in MCF-7 cells and tested whether disruption of TM4SF1 in HR+/HER2-breast cancer cells affects the cell proliferation. As shown in Figures 5A,B, TM4SF1 mRNA and protein levels decreased obviously in MCF-7 after being transfected with TM4SF1-siRNA. Consequently, cells transfected with TM4SF1-siRNA grow slower than control cells in the MTT assay (Figure 5C). However, TM4SF1-siRNA transfection results in the opposite phenomenon in the triple-negative breast cancer (ER-/PH-/HER2-) cell line MDA-MB-231 (Figures 5D–F).

### TM4SF1 Overexpression MCF-7 and ZR-75-1 Regulates Tumor Growth *In Vivo*

**FIGURE 6:**
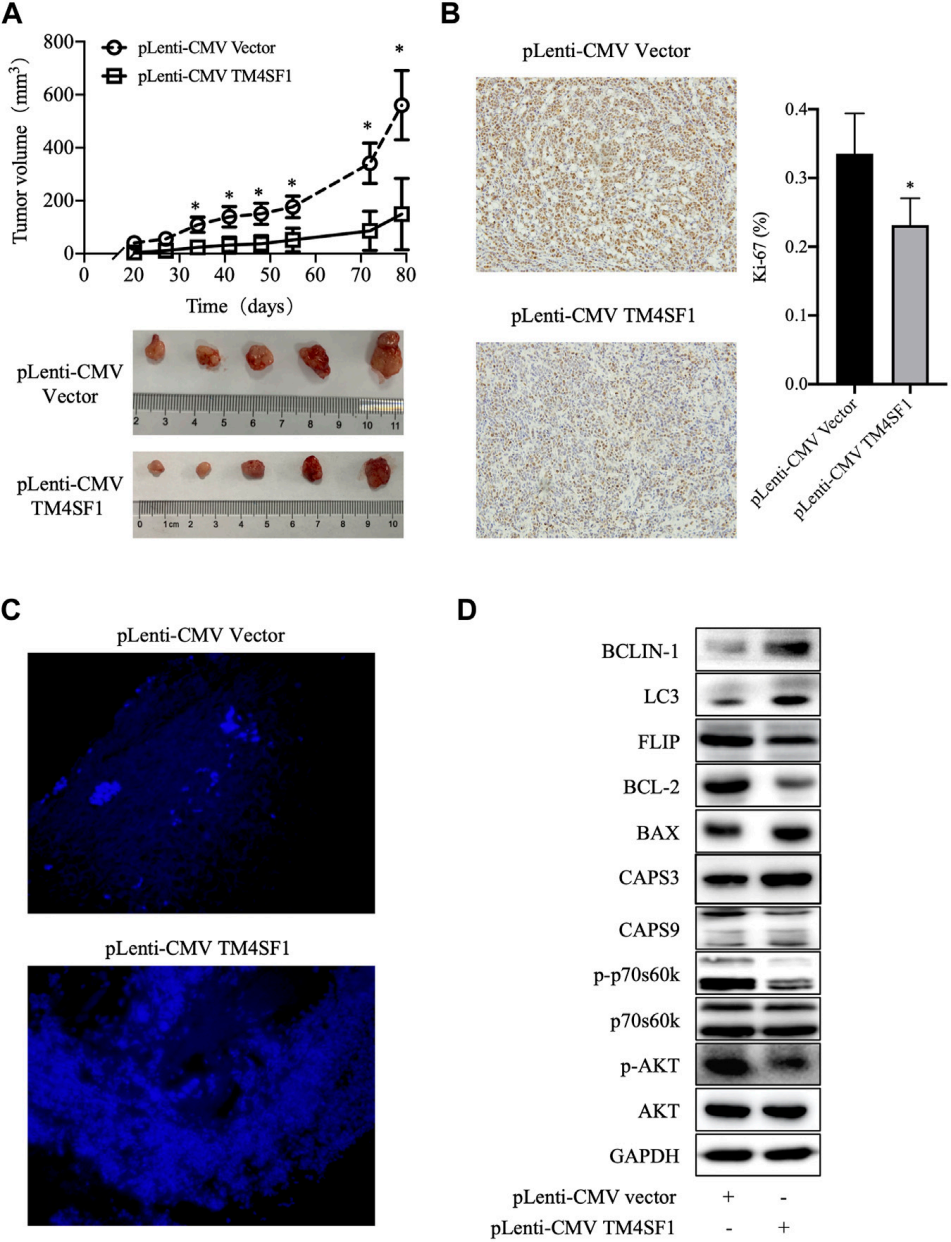
TM4SF1 overexpression inhibits the growth of MCF-7 xenografts. **(A)** Tumor growth curve of MCF-7 cells transfected with pLenti-CMV TM4SF1 or pLenti-CMV vectors (upper panel). Tumor images (lower panel). **(B)** Representative Ki-67 immunohistochemical staining image (left). Frequency of Ki-67^+^ cells (right, mean ± SD). ^*^ *p* < 0.05 compared with the pLenti-vector transfected group by *t*-test analysis. **(C)** TM4SF1 overexpression induces apoptosis in MCF-7 xenografts. Representative TUNEL staining images of apoptotic cancer cells are shown. **(D)** Effect of the TM4SF1 overexpression on the mTOR pathway, apoptosis, and autophagy in MCF-7 cells. GAPDH was used as a loading control. Data shown are representative of at least two independent experiments. TM4SF1, transmembrane 4 L six family 1.

Because the TM4SF1 expression was downregulated in HR^+^HER2^-^ breast cancer and the TM4SF1-overexpression inhibited MCF-7 and ZR-75-1 cell viability and proliferation *in vitro*, the roles of TM4SF1 in the HR^+^HER2^-^ breast tumor growth were examined in a murine model. As shown in Figure 6A, TM4SF1-overexpressing MCF-7 xenografts grew slower than those in the pLenti-CMV vector control group. Immunohistochemical staining data showed that the frequency of Ki-67^+^ cells was markedly reduced following the TM4SF1 overexpression (Figure 6B), indicating reduced proliferation. Furthermore, TUNEL immunofluorescence staining demonstrated that the TM4SF1 overexpression induced apoptosis *in vivo* (Figure 6C).

In addition, as shown in Figure 6D, the TM4SF1 overexpression downregulated the levels of phosphorylated AKT and phosphorylated p70s6k1, which indicated that TM4SF1 may inhibit the AKT/mTOR pathway. Consistent with TUNEL staining, cleaved caspase 3/9 and Bax protein levels were upregulated, while those of BCL-2 and FLIP were downregulated following the TM4SF1 overexpression. These results indicated that the TM4SF1 overexpression promoted apoptosis.

## Discussion

Breast cancer is the most common invasive cancer in women and the second leading cause of cancer death in women, after lung cancer (Parada et al., 2019). Moreover, although breast cancer is perhaps the most studied malignancy to date, the heterogeneity of this disease represents a major challenge for treatment (Piccart et al., 2021). Breast cancer is divided into different types depending on its origin, and the prognosis and treatment options for each type are generally based on the tumor-node-metastasis staging, lymphovascular spread, histological grade, HR status, ERBB2 (formerly HER2 or HER2/neu) overexpression, comorbidities, menopausal status, and age (Onitilo et al., 2009). In the present study, TM4SF1 was showed to be downregulated in HR^+^HER2^-^ breast cancer tissue samples, which suggested that it might act as a tumor suppressor for this breast cancer subtype.

However, most studies indicated that the high expression of TM4SF1 is correlated with the T stage, TNM stage, and lymph node metastasis in various cancer types, including prostate (Allioli et al., 2011), ovarian (Gao et al., 2019), glioma (Wang et al., 2015), colorectal (Park et al., 2017), liver (Zhu et al., 2021), thyroid (Lee et al., 2019), lung (Ma et al., 2018), pancreatic (Cao et al., 2016), and breast cancers (Tu et al., 2012; Xing et al., 2017; Fan et al., 2019). However, the low expression of TM4SF1 has been found to be associated with carcinogenesis and development, tumor progression, and invasion of gastric cancer (Peng et al., 2018), which indicates TM4SF1 is a tumor suppressor for gastric cancer and a novel prognostic marker for patients with gastric cancer. Thus, the role of TM4SF1 in cancer progression and invasion may also be tissue-specific.

The previous studies about TM4SF1 in breast cancer progression and development were also controversial cause utilizing different cell lines randomly (Abba et al., 2004; Simpson et al., 2010; Gao et al., 2016). A comparative SAGE analysis of mammary ductal carcinoma *in situ* (DCIS) versus normal breast epithelium revealed that the expression of TM4SF1 is significantly downregulated in DCIS (Abba et al., 2004). In the MCF-7 breast cancer cell line, the overexpression of p23 results in increased invasion, which is associated with TM4SF1 downregulation (Simpson et al., 2010). However, in TNBC cell lines 4T1 and MDA-MB-231 cells, TM4SF1 promotes cancer stem cell traits mechanistically by coupling DDR1 to PKCα and augmenting JAK-STAT signaling. In the present study, MCF-7 and ZR-75-1 cells (which are ER^+^PR^+^HER2^-^ cell lines) were used to investigate the role of TM4SF1 in HR^+^HER2^-^ breast cancer. The results demonstrated that the overexpression of TM4SF1 in these cell lines resulted in a decrease in their viability, 3D organoid formation, and colony formation. Conversely, TM4SF1 siRNA transfection increased the MCF-7 cell proliferation. By contrast, in the MDA-MB-231 TNBC cell line where TM4SF1 is overexpressed, TM4SF1 siRNA decreased the MDA-MB-231 cell proliferation. *In vitro,* TM4SF1-overexpressing MCF-7 xenografts grew at a slower rate than the control group. In tumor xenograft tissue, the frequency of Ki-67^+^ cells was significantly reduced following the TM4SF1 overexpression, whereas TUNEL staining was increased. Consistent with these findings, cleaved caspase 3/9 and Bax protein levels were upregulated, while those of BCL-2 and FLIP were downregulated following the TM4SF1 overexpression. These results suggested that the TM4SF1 overexpression could inhibit cell proliferation and induces apoptosis.

Moreover, it was also shown that the phosphorylation of AKT and p70S6K1 levels was reduced following the TM4SF1 overexpression. By contrast, the levels of the autophagy markers Beclin one and LC3 were increased (Figure 6D). Excessive autophagy can result in cell death (Lupinacci et al., 2021). Increased apoptosis was also observed in TM4SF1-overexpressing cells. These findings indicate that TM4SF1 may be involved in HR^+^HER2^-^ breast cancer development *via* the mTOR pathway.

In conclusion, TM4SF1 mRNA and protein levels were downregulated in HR^+^HER2^-^ breast cancer compared with noncancerous tissue samples. The overexpression of TM4SF1 inhibited breast cancer growth *in vivo*, as well as breast cancer cell viability and proliferation *in vitro*. Thus, TM4SF1 suppresses the HR^+^HER2^-^ breast cancer cell proliferation, but the exact role of this molecule remains unclear and deserves further investigation.

## Data Availability

The raw data supporting the conclusion of this article will be made available by the authors, without undue reservation.
